# Supplementary material for: Causal Relationship between Aging and Anorexia Nervosa: A White-Matter-Microstructure-Mediated Mendelian Randomization Analysis
Source: Biomedicines. 2024 Aug 16;12(8):1874. doi: 10.3390/biomedicines12081874 (PMC11351342; doi:10.3390/biomedicines12081874)
Supplement: Supplementary file 1 [file biomedicines-12-01874-s001.zip › Supplementary Figure/Figure S8/Leaveoneout plot of MR analysis for TL to ubm-b-2098.pdf]

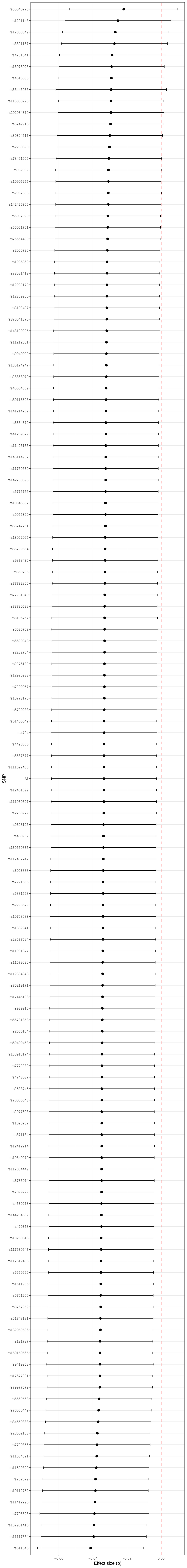

rs35640778
rs1291143
rs17803849
rs3891167
rs4731541
rs16978028
rs4616688
rs35446936
rs116863223
rs202034370
rs5742915
rs80324517
rs2230590
rs78491606
rs932002
rs10905255
rs2967355
rs142426306
rs6007020
rs56061761
rs75664430
rs2056726
rs1985369
rs73581419
rs12932179
rs12369950
rs8102497
rs376641875
rs143190905
rs11212631
rs9940099
rs185174247
rs28363070
rs45604339
rs80116508
rs141214782
rs6584579
rs41269079
rs11426156
rs145114957
rs11769630
rs142730696
rs6776756
rs10845387
rs9955360
rs55747751
rs13062095
rs56799554
rs9878436
rs869785
rs77732866
rs77231040
rs73730598
rs8105767
rs6536702
rs6590343
rs2282764
rs2276182
rs12925933
rs7209057
rs10773176
rs6790988
rs61405042
rs4724
rs4498805
rs6587577
rs111527438
All
rs12451892
rs111950327
rs2763979
rs9398196
rs450962
rs139669835
rs117407747
rs3093888
rs7221585
rs6881568
rs2293579
rs10768683
rs1332941
rs28577594
rs11991877
rs11579626
rs112394943
rs76219171
rs17445108
rs939916
rs66731853
rs2555104
rs59409453
rs188918174
rs7772289
rs4743037
rs2538745
rs76065543
rs2977608
rs1023767
rs871134
rs12412214
rs10840270
rs117034449
rs3785074
rs7099229
rs4530278
rs144204502
rs429358
rs13230646
rs117630647
rs117512405
rs6659669
rs1611236
rs6751209
rs3767952
rs61748181
rs182059586
rs131797
rs150150565
rs9419958
rs17677991
rs79977579
rs6669563
rs76666449
rs34550383
rs28502153
rs7790856
rs11584821
rs11699829
rs762679
rs10112752
rs11412296
rs7705526
rs137901416
rs11117354
rs611646
SNP
-0.06
-0.04
-0.02
0.00
Effect size (b)
